# Low-Level PM_2.5_ Exposure and Mortality in the Medicare Cohort: The Role of Native American Beneficiaries

**DOI:** 10.3390/ijerph22091340

**Published:** 2025-08-27

**Authors:** Judy Wendt Hess, Wenyaw Chan

**Affiliations:** 1 Hess Epidemiology Services, LLC, Houston, TX 77018, USA; 2 Department of Biostatistics and Data Science, University of Texas Health Science Center at Houston, Houston, TX 77030, USA; wenyaw.chan@uth.tmc.edu

**Keywords:** fine particulate matter, mortality, Medicare, American Indian or Alaska Native

## Abstract

Fine particulate matter (PM_2.5_) has been associated with mortality at low concentrations, with higher per-unit risk at lower exposure levels, and no threshold of effect. We examined characteristics of Medicare decedents living in zip codes at the lowest end of the U.S. PM_2.5_ exposure distribution to determine whether there is a demographic, health or exposure profile of beneficiaries for whom even low PM_2.5_ exposure is associated with increased mortality. The study included 2,773,647 decedent cases and 27,736,470 non-decedent controls, matched on decile of long-term PM_2.5_ exposure from among 36 million Medicare fee-for-service beneficiaries enrolled 2015–2016. Outcomes of the study included all-cause and cause-specific mortality, stratified by decile and beneficiary characteristics. Increased PM_2.5_-related mortality within the lowest exposure decile was found only among Native American beneficiaries, with odds ratios of 1.11 (95% CI, 1.01–1.21) and 1.21 (95% CI, 1.11–1.32) per 1 µg/m^3^ increase in PM_2.5_, for those eligible and ineligible for Medicaid, respectively, and was driven by significant increases in selected kidney and cardiovascular outcomes, diabetes, and chronic obstructive pulmonary disease. These results may reflect particular sensitivity to PM_2.5_; factors varying with PM_2.5_ at the zip code level, including constituent exposures or social determinants of health; or inaccuracies in exposure estimates.

## 1. Introduction

Recent research on long-term PM_2.5_ and mortality has focused on low-level exposures [1]. Much of this research in the U.S. was conducted with the Medicare cohort, which consistently found that even as ambient PM_2.5_ concentrations continued to fall, significant associations between PM_2.5_ and mortality persisted [2,3,4,5,6]. No threshold concentration has been identified in these studies below which a mortality association is not observed. A significant 2% increase in mortality was reported at average PM_2.5_ concentrations of 6.60 µg/m^3^ compared to the lowest exposure decile (4.35 µg/m^3^), and an additional doubling of risk for those with average exposure of 7.78 µg/m^3^ [5]. When the distribution was divided into 14 bins rather than 10 deciles, the pattern remained the same, with a hazard ratio (HR) of 1.02 for beneficiaries with average exposure of 5.77 µg/m^3^ compared to the lowest exposure bin (3.83 µg/m^3^). All of these concentrations are well below the U.S. EPA (EPA) annual PM National Ambient Air Quality Standard (NAAQS) of 9 µg/m^3^ [7].

Additionally, a higher per-unit risk has been found at lower exposure levels [3,8,9]. Di et al. reported an HR of 1.07 for each 10 µg/m^3^ increase in PM_2.5_ in their full analysis compared to 1.14 when only person-years exposed below the NAAQS at the time (12 µg/m^3^) were included, reflecting a steeper slope of the concentration response function (CFR) below this exposure level [3]. These authors observed significant increases in mortality at concentrations as low as 5 µg/m^3^, and no evidence of a threshold.

CFRs reflect relative risk adjusted for covariates that might otherwise bias estimates. However, controlling for these covariates precludes an understanding of the population segment driving the observed effect. Our objective was to examine characteristics of Medicare decedents living in zip codes at the lowest end of the PM_2.5_ exposure distribution to determine whether there is a demographic, health or exposure profile of beneficiaries for whom even low PM_2.5_ exposure is associated with increased risk of death. For this reason, our results will focus on the lowest exposure decile (“decile 1”), which was defined as a 365-day average PM_2.5_ concentration less than or equal to 4.68 µg/m^3^.

## 2. Materials and Methods

### 2.1. Medicare Data

We obtained Medicare data from the Centers for Medicare and Medicaid Services (CMS) including the Master Beneficiary Summary File (MBSF) Base, MBSF 27 Chronic Conditions Segment, and MBSF National Death Index Segment annual files for 2015 and 2016. The files included person-level records of all Medicare beneficiaries 65 years of age or older living in the contiguous U.S. and enrolled in a traditional fee-for-service Medicare program for the entire calendar year. The files included age, sex, race, residential zip code, dual Medicare/Medicaid eligibility, history of chronic health conditions, and date and cause(s) of death. Beneficiaries eligible for Medicaid benefits during any month of the study period were considered dual-eligible in the analysis.

The chronic conditions segment contained person-level history of past or current diagnosis of 27 conditions based on medical encounter records. For each decedent, the National Death Index segment contained an International Classification of Diseases Tenth Revision (ICD-10) underlying cause-of-death code, and an ICD-10 recode reflecting 113 selected causes of death for each decedent [10].

### 2.2. Ambient Air Pollution Data

Daily average concentrations of ambient PM_2.5_, nitrogen dioxide (NO_2_), and ozone (O_3_) were obtained from the NASA Socioeconomic Data and Applications Center (SEDAC) for 2015 and 2016 [11]. Methods used to derive these estimates have been described in detail elsewhere [12]. In brief, pollutant concentrations were predicted at the centroid of 1 km by 1 km grid cells across the contiguous U.S. using a series of machine-learning models incorporating ground and satellite monitoring data, meteorological and land use variables, and chemical transport models. These predicted values were used to estimate daily exposure at the zip code level and have been employed in previous Medicare studies [5,6]. We used daily pollutant estimates to calculate average exposure in the preceding 365 days (i.e., average annual exposure), for each zip code represented in the Medicare beneficiary files, on each day of 2015 and 2016. From these values, deciles of annual average PM_2.5_ were calculated based on the full 2-year distribution, and assigned to each zip code, on each day of 2015 and 2016. Three short-term exposure variables were also defined for each pollutant on the day of death (L0), and one (L1) and two (L2) days preceding death.

### 2.3. Covariate Data

Files containing estimated maximum and minimum outdoor temperature at 1 km by 1 km grid cells across the contiguous U.S., for each day of 2015 and 2016, were produced by the Environmental Sciences Division at the Oak Ridge National Laboratory and obtained from the NASA Earth Science Data and Information System [13]. We used ESRI ArcGIS to assign daily zip code-level temperature values based on the 1 km grid values estimated at the centroid of each zip code.

Social Vulnerability Index (SVI), developed by the U.S. Centers for Disease Control and Prevention (CDC), was obtained from SEDAC [14]. We used ESRI ArcGIS to convert 2016 SVI values estimated at 1 km by 1 km grid cells across the contiguous U.S. to zip code-level values by identifying the SVI value at the centroid of each zip code.

Urban-rural classification for each zip code was obtained from the USDA Economic Research Service 2010 Rural-Urban Commuting Area (RUCA) Codes [15]. Primary RUCA codes were dichotomized into metropolitan (values 1–3) or non-metropolitan (values 4–10).

Zip code-level indicators of socioeconomic status (SES) were obtained from the U.S. Census Bureau 2010 Decennial Census (percent white, black, Hispanic and North American Native (NAN) residents; population density), and 2011–2015 American Community Survey (percent of residents with less than a high school education; percent of owner-occupied homes; percent of residents 65 and older living below the poverty level; median household income; median value of owner-occupied housing) [16].

### 2.4. Statistical Analysis

In this case–control study, all beneficiaries who died in 2015 or 2016 were selected as cases and assigned decile of annual average PM_2.5_ exposure based on their zip codes and dates of death as described above. Ten controls per case were selected from among all beneficiaries alive on the case’s date of death, matched on decile of exposure at the control’s zip code on the case’s date of death. For example, for a decedent whose zip code at death was within decile 5, the universe of zip codes assigned to decile 5 on the case’s date of death were identified, and controls were randomly selected from among those residing in one of these zip codes. Beneficiaries were excluded if their zip code was either missing or not contained in the PM_2.5_ files, as a decile of exposure could not be assigned (Figure S1).

We used unconditional logistic regression to estimate all-cause and cause-specific odds ratios (OR) per 1 µg/m^3^ increase in PM_2.5_ within deciles, and conditional logistic regression to estimate ORs for all deciles combined. After preliminary analysis, the following variables were retained in logistic regression models: age, sex, race, dual-Medicaid eligibility; and zip code-level annual average NO_2_ and O_3_, SVI, metropolitan residence, percent white, black, Hispanic and NAN residents; percent of residents with less than a high school education; percent of residents 65 and older living below the poverty level; and percent of owner-occupied homes. Month of case’s death (1–24) was included in decile-specific analyses, and stratified analyses by Medicaid eligibility and race were also conducted. The outcome variable for cause-specific analyses was the 113 ICD-10 cause of death recode. Results are presented only for causes with 11 or more cases.

Analyses were performed using SAS 9.4, and SAS Enterprise Guide software (SAS Institute, Inc., Cary, NC, USA). Zip code-level meteorological and SVI data were generated with ArcGIS Pro, version 3.4.0 (Esri, Redlands, CA, USA). The study was approved by the Centers for Medicare and Medicaid Services Privacy Board and the University of Texas Health Science Center Committee for the Protection of Human Subjects.

## 3. Results

### 3.1. Characteristics of Study Participants

From among 36,080,439 unique beneficiaries, 2,773,647 decedent cases and 27,736,470 non-decedent controls were selected for the study (Table 1). Cases were older on average than controls (82.1 years vs. 74.9 years, respectively), with a slightly higher proportion of males (46.7% vs. 45.4%, respectively). A greater proportion of cases were white, black and NAN (86.2% vs. 83.7%; 8.7% vs. 8.3%; 0.5% vs. 0.4%, respectively), and a smaller proportion were Hispanic and Asian (1.7% vs. 1.9%; 1.5% vs. 2.2%, respectively). Zip code-level measures from the Census Bureau indicated lower SES for cases vs. controls, which was consistent with the proportion of each who were Medicaid-eligible (27.9% vs. 13.2%, respectively).

Compared to cases overall, a greater proportion of decedents in decile 1 were male, white and NAN (50.1% vs. 46.7%; 92.2% vs. 86.2%; 3.1% vs. 0.5%, respectively); and a lower proportion were black and Medicaid-eligible (1.0% vs. 8.7%; 24.1% vs. 27.9%, respectively) (Table 1). The proportion of white and NAN decedents, and zip code-level income and education levels decreased with increasing decile, while the proportion of black and Medicaid-eligible decedents, and proportion of deaths increased across deciles (Table S1).

Annual average PM_2.5_ exposure for cases and controls across all deciles was 7.81 µg/m^3^, and in decile 1, averaged 3.70 µg/m^3^ and 3.67 µg/m^3^ for cases and controls, respectively (Table 2). Exposure was lowest for NAN beneficiaries, particularly among those eligible for Medicaid. Median PM_2.5_ in decile 1 was 3.81 µg/m^3^ compared to 3.26 µg/m^3^ for NANs, meaning that PM_2.5_ exposure at their zip codes of residence clustered at the lower end of the lowest decile (Table S2).

### 3.2. Mortality

PM_2.5_ was positively associated with all-cause mortality within lower deciles, including decile 1 (adjusted OR, 1.013; 95% CI, 1.005–1.022), but not within upper deciles (Table 3 and Table S3). When stratified by race, the increase in decile 1 was limited to NANs, including those eligible (OR, 1.105; 95% CI, 1.006–1.213) and ineligible (OR, 1.213; 95% CI, 1.112–1.324) for Medicaid (Table 3). When NANs were excluded from the adjusted analysis, the association with PM_2.5_ was attenuated and no longer statistically significant (Table S3).

Among NANs in decile 1, PM_2.5_ was significantly associated with death from kidney cancer (OR, 3.01; 95% CI, 1.06–8.56), diabetes (OR, 1.53; 95% CI, 1.12–1.96), acute myocardial infarction (OR, 1.41; 95% CI, 1.02–1.95), chronic obstructive pulmonary disease (COPD, OR, 1.78; 95% CI, 1.32–2.39), and renal failure (OR, 1.86; 95% CI, 1.21–2.83); and elevated but not significant for lung cancer (OR, 1.37; 95% CI, 0.998–1.87, Table 4). A grouped category for overall cardiovascular disease [17] (ICD10: I20–I25, I50) was also elevated but not significant (OR, 1.17; 95% CI, 0.990–1.39).

**Figure 1:**
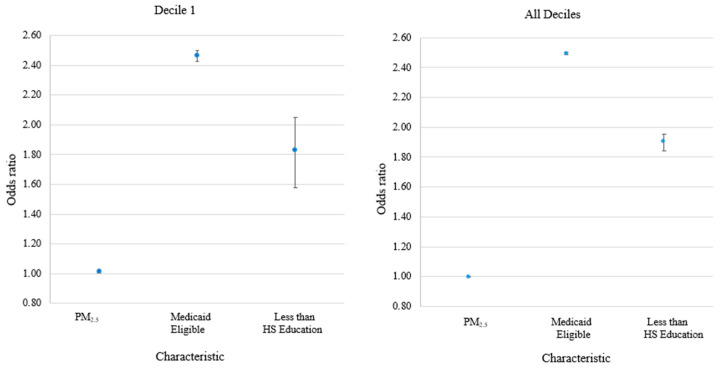
Associations of PM_2.5_ and socioeconomic status indicators with all-cause mortality, decile 1 and all deciles combined. Odds ratios reflect increase in all-cause mortality associated with each 1 µg/m^3^ increase in average PM_2.5_ in the 365 days preceding cases’ death dates, increase in all-cause mortality for Medicaid eligible vs. ineligible beneficiaries, and increase in all-cause mortality for each 1 percentage point increase in the zip code level proportion of residents with less than a high school education. Error bars represent 95% confidence interval. Conditional logistic regression models for All Deciles were adjusted for age, sex, race, dual-Medicaid eligibility; zip code-level annual average NO_2_ and O_3_, SVI, metropolitan residence, percent white, black, Hispanic and Native American residents; percent of residents with less than a high school education; percent of residents 65 and older living below the poverty level; percent of owner-occupied homes. Unconditional logistic regression models for Decile 1 also included month of case’s death.

SES was a stronger predictor of all-cause mortality than PM_2.5_ (Figure 1). ORs for Medicaid eligibility were 2.47 (95% CI, 2.43–2.50) and 2.49 (95% CI, 2.49–2.50) for decile 1 and all deciles, respectively, and for each percentage point increase in the proportion with less than a high school education were 1.83 (95% CI, 1.61–2.08) and 1.90 (95% CI, 1.85–1.96), respectively.

## 4. Discussion

This study explored the persistent finding that higher PM_2.5_ exposure well below the current PM NAAQS increases mortality without any threshold, and with higher per-unit risk at lower concentrations. We found that among the lowest-exposed Medicare beneficiaries, NANs were the only subgroup whose risk of death increased with PM_2.5_, despite having the lowest average exposure. There are several possible explanations for our findings. Social determinants of health (SDOH) likely play a role. NANs may be uniquely sensitive to the effects of PM_2.5_ due to longstanding economic, social and health disparities. There may also be SDOH, such as healthcare access, that vary with PM_2.5_ at the zip code or even state level [18]. Housing characteristics, including poorly constructed homes, insufficient ventilation and use of wood-burning stoves may exacerbate personal PM_2.5_ exposure, even in the presence of relatively low ambient concentrations [19,20,21]. Exposure to toxic PM_2.5_ constituents that co-vary with aggregate PM_2.5_ at the zip code level could also explain our findings. And finally, predicted PM_2.5_ concentrations at the zip code level may underestimate exposure in the western U.S. where most decile 1 NAN beneficiaries lived.

SDOH are at their core about access—to nutritious food, clean air, safe and affordable housing, quality education and healthcare, employment opportunities, and secure and thriving communities [22]. Inequities across these domains, and the resulting health impacts among NANs, have spanned generations [23]. NANs have significantly higher mortality compared to white, black and Hispanic populations in the U.S. overall, for most leading causes of death, and at most ages, including the elderly [24]. Their life expectancy at birth is 4–10 years less than other populations [24]. They have higher rates of obesity, heart disease, diabetes, and substance use disorders, and lower rates of physical activity, are twice as likely to perceive their health as poor or fair, and more likely to be disabled or uninsured [25,26]. The prevalence of cigarette smoking among NAN adults is among the highest in the U.S. and has declined to a lesser degree over time compared to other racial groups [27,28]. The impact of possibly decades of smoking on heart, lung and kidney health, as well as chronic disease rates, may heighten their sensitivity to air pollution exposure [29]. The Indian Health Service (IHS), tasked by the federal government to provide medical and public health services to NAN tribes, is plagued by a lack of funding, provider shortages and aging facilities, all of which constrain their ability to deliver care in the often sparsely populated areas they serve [30]. Across the U.S., the quality and availability of care delivered through the IHS varies by location, type of facility, and availability of supplemental funding sources [31]. Medicaid eligibility requirements also vary by state [32].

Lower SES individuals are more susceptible to the effects of air pollution [33]. SES also mediates air pollution-related health risk, because lower-income communities are often more highly exposed [34]. Nearly a third of NANs live in poverty, with this proportion exceeding 60% among some tribes [35]. In our analysis, nearly all SES indicators pointed to their relative economic disadvantage compared to other beneficiaries, with the exception of dual Medicaid eligibility, the only person-level SES indicator in the Medicare files (Table S4). SES is strongly predictive of mortality in the Medicare cohort; Medicaid eligibility and lower educational attainment increased the risk of death nearly two to three-fold in every decile.

Point source emissions of toxic particle constituents or other environmental hazards that correlate with PM_2.5_ could also explain our findings. Ambient PM_2.5_ concentrations declined U.S.-wide between 2000 and 2018, but improvements in counties heavily populated by NANs lagged the rest of the nation [36]. The same trend was observed for PM_2.5_ constituents, especially ammonium and sulfate [37]. A 2024 analysis found that emissions of ammonia, nitrogen oxides, and sulfur dioxide from the agriculture, energy and industry sectors, respectively, increased to a greater extent in counties experiencing growth in NAN residents [38]. “Coal PM_2.5_” is also associated with over twice the mortality risk compared to aggregate PM_2.5_ [39], an exposure that disproportionately impacts NANs [40].

Finally, PM_2.5_ exposure among NANs concentrated in sparsely populated western states may be underestimated by models used to predict zip-code level concentrations in this and other Medicare studies. Model performance was degraded in the “mountain” region including Arizona and New Mexico [12], where 62% of NANs in decile 1 lived (Table S5). Other models have been proposed to accommodate the complex terrain of this area, which includes mountains, valleys, dust storms, wildfires and snow cover [41]. In reality, little monitoring data exists to validate ambient predictions on U.S. tribal lands. Three EPA monitoring sites collected PM_2.5_ samples on tribal lands in Arizona in 2015–2016, and six others in nearby rural areas [42]. Although this provides only a limited sample in space and time, a comparison of measured and predicted values at monitoring site zip codes revealed significant gaps, particularly for high exposure days [42] (Table S6).

We are not aware of prior studies presenting mortality estimates for race-specific strata, within the lowest subsets of the PM_2.5_ distribution. Previous Medicare studies have not evidenced the role of NAN beneficiaries, although they did report a greater proportion at low exposures [3,4,43]. Di et al. reported race-specific mortality risk [for NANs, HRs ranged from 1.100 (95% CI, 1.060–1.140) to 1.145 (95% CI, 1.090–1.203) depending on the model], and risk estimates restricted to exposures below 12 µg/m^3^, but they did not report race-specific estimates in the low-exposure group [3]. A later update did not provide race-specific results [4]. Wei et al. categorized race as white, black or other and did not report race-stratified results [5], and other studies excluded NANs from reported results altogether [44,45,46]. All studies appropriately adjusted for race; however, this precluded an assessment of effect modification by decile and race.

Cardiovascular, and to a lesser extent respiratory and neurological diseases, have been causally linked to long-term PM_2.5_ exposure by the EPA [29]. Other evidence supports broader health impacts. Agreement between our results and recent Medicare [47] and Veteran’s Administration [17] studies was mixed. We confirmed associations of PM_2.5_ with diabetes, COPD and myocardial infarction, but not with death from heart disease overall, heart failure, cerebrovascular disease, hypertension, dementia or pneumonia among NANs in decile 1. We found increases in renal failure and kidney cancer, despite a small number of cases. Many of these causes of death have been associated with PM_2.5_, but also with health and behavioral risk factors prevalent among NANs [48,49,50], particularly among those living in rural areas [51] and tribal lands [52].

Our study had several limitations. The accuracy of race coding in the MBSF is variable and particularly low for NANs [53,54], although this has improved since 1999 due to IHS data-sharing [55]. Our analysis included over 30 million beneficiaries, but fewer than 3 million decedents, and stratification resulted in small population subsets, particularly in cause-specific analysis. We also acknowledge that the data used in our analysis are nearly a decade old. We chose to use 2015–2016 data in order to align with 2000–2016 cohort and exposure data used in recent Medicare mortality studies. These studies consistently observed no effect threshold and a steeper slope at the lowest end of the concentration response curve, and we sought to replicate, and then expand upon, this work. While we believe more recent data is needed to understand the relationship between PM_2.5_ and mortality at present-day ambient concentrations, these studies continue to inform current understanding of the risk of PM_2.5_, and their low-level concentrations are still relevant today. There was also potential redundancy introduced into our models by including both SVI and two of our SES covariates which also comprise the summary SVI, specifically the proportion of zip code residents by race, and with less than a high school education. SVI components not included as separate covariates in our models addressed factors such as unemployment, the cost of housing, health insurance coverage, disabilities, single parent households, English language proficiency and housing characteristics. In our analysis, model performance was improved when both SVI and the two specific components were included, likely due to the diverse measures of vulnerability captured by the SVI. Finally, PM_2.5_ exposure was estimated only at the residential zip code level, and person-level covariate information, which could potentially explain variability in risk across deciles, Medicaid eligibility and race, is largely unavailable for Medicare beneficiaries. No person-level smoking or alcohol consumption data were available in the Medicare files used in our analysis. However, earlier Medicare air pollution and mortality studies encountered these same data constraints.

NANs comprised just 0.5% of Medicare decedents in 2015–2016, but 3.1% of decile 1 deaths, second only to whites. Over a third of NAN decedents lived within decile 1 zip codes, and nearly half within the lowest 20% of the distribution. Our analysis provides evidence that NANs may be an underappreciated driver of the association between mortality and low-level PM_2.5_ exposure in the Medicare cohort. Whether particular vulnerability to PM_2.5_ or another factor that co-varies with PM_2.5_ explains our results, it is doubtful that a threshold will be observed in the Medicare cohort so long as this population, characterized by low SES and high rates of chronic disease and mortality, is clustered at the bottom of the distribution, regardless of how low absolute exposure levels become.

## 5. Conclusions

Both public and private sector policies and practices have contributed to the profound health disparities evident among NANs [56]. Further study is needed to elucidate drivers of our findings, which can then inform effective mitigations that address these disparities without unnecessarily worsening community-level economic wellbeing.

## Figures and Tables

**Table 1:** Comparison of study population characteristics, decile 1 vs. all deciles.

Characteristic	Decile 1, No. (%)		All Deciles, No. (%)	
	Cases (n = 153,781)	Controls (n = 1,537,810)	Cases (n = 2,773,647)	Controls (n = 27,736,470)
**Beneficiary-level covariates**				
Age, mean (SD), y	81.8 (9.3)	74.4 (7.6)	82.1 (9.3)	74.9 (8.1)
Sex				
Male	76,972 (50.1)	744,196 (48.4)	1,294,125 (46.7)	12,577,617 (45.4)
Female	76,809 (50.0)	793,614 (51.6)	1,479,522 (53.3)	15,158,850 (54.7)
Race				
White	141,834 (92.2)	1,400,476 (91.1)	2,391,249 (86.2)	23,222,339 (83.7)
Black	1589 (1.0)	15,444 (1.0)	240,845 (8.7)	2,293,857 (8.3)
Other	1269 (0.8)	19,161 (1.3)	28,146 (1.0)	480,618 (1.7)
Asian	926 (0.6)	11,859 (0.8)	40,284 (1.5)	604,350 (2.2)
Hispanic	2732 (1.8)	24,296 (1.6)	45,728 (1.7)	515,222 (1.9)
Native American	4709 (3.1)	39,457 (2.6)	14,217 (0.5)	122,238 (0.4)
Unknown	722 (0.5)	27,117 (1.8)	13,178 (0.5)	497,846 (1.8)
Dual Medicaid Eligibility	37,013 (24.1)	165,480 (10.8)	773,515 (27.9)	3,669,176 (13.2)
**Zip code-level covariates**				
Urbanicity				
Metropolitan	72,010 (46.8)	746,108 (48.5)	2,109,954 (76.1)	21,704,297 (78.3)
Micropolitan	36,708 (23.9)	351,464 (22.9)	342,613 (12.4)	3,113,969 (11.2)
Small Town	22,790 (14.8)	216,571 (14.1)	191,153 (6.9)	1,704,315 (6.1)
Rural	22,247 (14.5)	223,441 (14.5)	129,772 (4.7)	1,212,823 (4.4)
Social Vulnerability Index, mean (SD)	0.45 (0.26)	0.43 (0.26)	0.45 (0.27)	0.43 (0.27)
Percent White	86.4	86.8	77.7	77.6
Percent Black	1.7	1.6	11.5	11.0
Percent Hispanic	14.9	14.6	12.7	13.0
Percent Native American	3.8	3.5	0.8	0.8
Median household income, USD	50,621	52,003	52,270	54,840
Median value owner-occupied housing, USD	205,300	217,900	170,900	183,000
Percent of elderly below poverty level	8.6	8.3	9.4	9.0
Percent w/less than HS education	11.1	10.6	12.6	12.1
Percent of owner-occupied housing	70.4	71.2	66.7	67.2
Population density, No.	433	441	2756	2966
Annual PM_2.5_, mean (SD)	3.70 (0.74)	3.67 (0.75)	7.81 (1.87)	7.81 (1.88)
Lag 1 ^1^ PM_2.5_, mean (SD)	3.71 (2.84)	3.67 (2.80)	7.64 (4.56)	7.63 (4.58)
Annual NO_2_, mean (SD)	11.00 (5.09)	11.07 (5.15)	15.63 (7.61)	15.93 (7.72)
Lag 1 ^1^ NO_2_, mean (SD)	10.52 (6.36)	10.55 (6.39)	15.48 (10.07)	15.71 (10.17)
Annual O_3_, mean (SD)	41.98 (5.46)	42.08 (5.54)	38.75 (3.48)	38.80 (3.59)
Lag 1 ^1^ O_3_, mean (SD)	41.98 (9.90)	42.07 (9.96)	38.85 (10.42)	38.90 (10.48)

^1^ Lag 1 represents daily PM_2.5_ concentration on the day prior to cases’ dates of death.

**Table 2:** Average annual ^1^ ambient PM_2.5_ exposure by case status, race and Medicaid eligibility.

Characteristic	Decile 1, µg/m^3^, Mean (SD)		All Deciles, µg/m^3^, Mean (SD)	
	Cases (n = 153,781)	Controls (n = 1,537,810)	Cases (n = 2,773,647)	Controls (n = 27,736,470)
All	3.70 (0.74)	3.67 (0.75)	7.81 (1.87)	7.81 (1.88)
Not Medicaid-eligible	3.69 (0.74)	3.66 (0.75)	7.78 (1.86)	7.78 (1.86)
White	3.69 (0.74)	3.67 (0.75)	7.72 (1.87)	7.71 (1.87)
Black	3.77 (0.78)	3.77 (0.80)	8.58 (1.46)	8.53 (1.45)
Hispanic	3.54 (0.76)	3.66 (0.76)	8.04 (2.32)	8.23 (2.16)
Native American	3.53 (0.67)	3.43 (0.65)	6.31 (2.15)	6.28 (2.18)
Medicaid-eligible	3.74 (0.72)	3.71 (0.73)	7.91 (1.87)	8.06 (1.95)
White	3.78 (0.72)	3.74 (0.73)	7.73 (1.86)	7.81 (1.93)
Black	3.76 (0.83)	3.83 (0.79)	8.55 (1.44)	8.59 (1.46)
Hispanic	3.70 (0.74)	3.78 (0.72)	8.28 (2.31)	8.43 (2.34)
Native American	3.34 (0.62)	3.27 (0.59)	5.77 (2.37)	5.57 (2.44)

^1^ Calculated as the mean of daily average PM_2.5_ concentrations for the 365 days prior to cases’ date of death.

**Table 3:** Risk of death associated with ambient PM_2.5_, by race and Medicaid eligibility.

Characteristic	Odds Ratio (95% CI) ^1^			
	Decile 1		All Deciles	
	Unadjusted	Adjusted	Unadjusted	Adjusted
All	1.060 (1.052–1.067)	1.013 (1.005–1.022)	1.003 (1.000–1.006)	0.998 (0.995–1.001)
Not Medicaid-eligible	1.043 (1.035–1.052)	1.012 (1.002–1.022)	1.000 (0.996–1.003)	0.998 (0.994–1.002)
White	1.047 (1.038–1.056)	1.009 (0.999–1.019)	1.004 (1.001–1.008)	0.997 (0.993–1.001)
Black	1.002 (0.923–1.088)	1.029 (0.934–1.133)	1.012 (0.985–1.040)	1.011 (0.978–1.044)
Hispanic	0.825 (0.762–0.894)	1.006 (0.907–1.116)	1.114 (1.008–1.231)	1.089 (0.937–1.267)
Native American	1.239 (1.158–1.327)	1.213 (1.112–1.324)	1.288 (1.003–1.654)	1.441 (1.020–2.038)
Medicaid-eligible	1.066 (1.050–1.083)	1.007 (0.988–1.026)	0.935 (0.929–0.941)	1.006 (0.999–1.014)
White	1.064 (1.045–1.082)	1.004 (0.984–1.025)	0.965 (0.955–0.974)	1.014 (1.002–1.025)
Black	0.893 (0.805–0.990)	0.893 (0.788–1.012)	0.966 (0.928–1.004)	0.986 (0.940–1.033)
Hispanic	0.864 (0.806–0.926)	0.978 (0.898–1.064)	0.981 (0.936–1.029)	1.010 (0.947–1.077)
Native American	1.231 (1.150–1.318)	1.105 (1.006–1.213)	1.064 (0.807–1.401)	1.110 (0.727–1.694)

^1^ Odds ratios reflect increase in risk associated with each 1 µg/m^3^ increase in average PM_2.5_ in the 365 days preceding cases’ death dates. Odds ratios within decile 1 were estimated using unconditional logistic regression. Odds ratios for all deciles combined were estimated using conditional logistic regression, matched by PM_2.5_ decile. “Decile 1” models adjusted for age, sex, race, dual-Medicaid eligibility; zip code-level annual average NO_2_ and O_3_, SVI, metropolitan residence, percent white, black, Hispanic and Native American residents; percent of residents with less than a high school education; percent of residents 65 and older living below the poverty level; percent of owner-occupied homes, and month of case’s death. Exceptions: models stratified by Medicaid status were not adjusted for dual-Medicaid eligibility, and models stratified by race were not adjusted for race. “All deciles” models adjusted for age, sex, race, dual-Medicaid eligibility; zip code-level annual average NO_2_ and O_3_, SVI, metropolitan residence, percent white, black, Hispanic and Native American residents; percent of residents with less than a high school education; percent of residents 65 and older living below the poverty level; and percent of owner-occupied homes. Exceptions: models stratified by Medicaid status were not adjusted for dual-Medicaid eligibility, and models stratified by race were not adjusted for race.

**Table 4:** Associations between specific causes of death and PM_2.5_, decile 1 overall and decile 1 restricted to Native Americans.

113 ICD-10 Cause of Death Recode	113 ICD-10 Cause of Death Recode Description	ICD-10 Code(s)	All		Native Americans	
			Cases	OR ^1^ (95% CI)	Cases	OR ^1^ (95% CI)
003	Certain other intestinal infections	A04, A07–A09	631	1.02 (0.89–1.17)	31	1.08 (0.46–2.54)
010	Septicemia	A40–A41	1570	1.10 (1.01–1.20)	85	1.32 (0.79–2.22)
018	Other and unspecified infectious and parasitic diseases	A00, A05, A20–A36, A42–A44, A48–A49, A54–A79, A81–A82, A85.0–A85.1, A85.8, A86–B04, B06–B09, B25–B49, B55–B99	344	1.01 (0.85–1.20)	14	5.82 (0.80–42.21)
021	Malignant neoplasm of esophagus	C15	885	0.98 (0.88–1.09)	13	1.49 (0.46–4.86)
022	Malignant neoplasm of stomach	C16	429	1.11 (0.94–1.30)	27	0.74 (0.32–1.73)
023	Malignant neoplasm of colon, rectum, and anus	C18–C21	2722	0.95 (0.90–1.01)	80	1.17 (0.74–1.84)
024	Malignant neoplasm of liver and intrahepatic bile ducts	C22	1068	1.04 (0.95–1.15)	59	1.53 (0.81–2.87)
025	Malignant neoplasm of pancreas	C25	2264	1.05 (0.98–1.13)	51	1.09 (0.62–1.92)
027	Malignant neoplasm of trachea, bronchus, and lung	C33–C34	7596	1.06 (1.02–1.10)	152	1.37 (0.998–1.87)
029	Malignant neoplasm of breast	C50	1922	9.97 (0.90–1.04)	36	0.81 (0.38–1.71)
032	Malignant neoplasm of ovary	C56	747	1.01 (0.90–1.14)	18	1.27 (0.34–4.78)
033	Malignant neoplasm of prostate	C61	2269	0.94 (0.87–1.01)	63	1.29 (0.66–2.52)
034	Malignant neoplasm of kidney and renal pelvis	C64–C65	775	0.96 (0.85–1.08)	35	3.01 (1.06–8.56)
035	Malignant neoplasm of bladder	C67	109	0.94 (0.85–1.04)	13	2.35 (0.55–9.98)
036	Malignant neoplasm of meninges, brain, and other parts of the central nervous system	C70–C72	704	0.93 (0.83–1.04)	12	0.35 (0.02–1.15)
039	Non-Hodgkin’s lymphoma	C82–C85	1187	0.99 (0.90–1.08)	29	1.17 (0.53–2.62)
040	Leukemia	C91–C95	1325	1.07 (0.98–1.17)	20	0.77 (0.29–2.06)
041	Multiple myeloma and immunoproliferative neoplasms	C88, C90	684	0.97 (0.86–1.10)	13	0.87 (0.19–3.88)
043	All other and unspecified malignant neoplasms	C17, C23–24, C26–C31, C37–C41, C44–C49, C51–C52, C57–C60, C62–C63, C66, C68–C69, C73–C80, C97	4067	1.02 (0.97–1.07)	103	0.92 (0.61–1.39)
044	In situ neoplasms, benign neoplasms, and neoplasms of uncertain or unknown behavior	D00–D48	979	0.99 (0.89–1.09)	22	0.33 (0.11–0.98)
045	Anemias	D50–D64	292	0.97 (0.80–1.18)	17	1.05 (0.17–6.40)
046	Diabetes mellitus	E10–E14	4233	1.06 (1.01–1.12)	367	1.53 (1.12–1.96)
048	Malnutrition	E40–E46	442	0.87 (0.74–1.03)	20	0.36 (0.09–1.38)
051	Parkinson’s disease	G20–G21	2152	1.01 (0.94–1.09)	60	0.95 (0.53–1.71)
052	Alzheimer’s disease	G30	8441	0.93 (0.90–0.97)	117	0.86 (0.59–1.26)
059	Acute myocardial infarction	I21–I22	5851	1.10 (1.06–1.15)	157	1.41 (1.02–1.95)
062	Atherosclerotic cardiovascular disease	I25.0	3589	0.86 (0.81–0.90)	95	1.08 (0.72–1.61)
063	All other forms of chronic ischemic heart disease	I20, I25.1–I25.9	10,966	1.02 (0.99–1.05)	252	1.12 (0.85–1.46)
067	Heart failure	I50	4243	1.10 (1.04–1.16)	80	0.91 (0.57–1.48)
068	All other forms of heart disease	I26–I28, I34–I38, I42–I49, I51	7712	1.03 (0.99–1.07)	125	1.12 (0.79–1.58)
069	Essential (primary) hypertension and hypertensive renal disease	I10, I12	1843	1.03 (0.95–1.11)	51	1.32 (0.73–2.38)
070	Cerebrovascular diseases	I60–I69	8334	0.99 (0.96–1.03)	207	0.95 (0.71–1.27)
078	Pneumonia	J12–J18	2711	1.12 (1.05–1.20)	135	1.00 (0.64–1.55)
084	Emphysema	J43	612	1.01 (0.89–1.15)	13	0.38 (0.09–1.72)
086	Other chronic lower respiratory disease	J44, J47	10,144	1.04 (1.00–1.07)	180	1.78 (1.32–2.39)
087	Pneumoconiosis and chemical effects	J60–J66, J68	96	0.97 (0.66–1.42)	11	--
088	Pneumonitis due to solids and liquids	J69	1236	0.97 (0.88–1.07)	52	1.63 (0.78–3.41)
089	Other diseases of respiratory system	J00–J06, J30–J39, J67, J70–J98	2247	1.01 (0.94–1.09)	112	0.84 (0.52–1.37)
094	Alcoholic liver disease	K70	640	0.99 (0.87–1.12)	56	0.78 (0.38–1.62)
095	Other chronic liver disease and cirrhosis	K73–K74	652	1.05 (0.93–1.19)	49	1.68 (0.82–3.46)
096	Cholelithiasis and other disorders of the gallbladder	K80–K82	298	1.00 (0.82–1.23)	14	3.03 (0.33–27.90)
100	Renal failure	N17–N19	2366	1.05 (0.98–1.12)	125	1.86 (1.21–2.83)
110	Symptoms, signs, and abnormal clinical and laboratory findings	R00–R99	1354	1.00 (0.91–1.11)	41	0.47 (0.19–1.19)
111	All other diseases	D65–E07, E15–E34, E65–F99, G04–G12, G23–G25, G31–H93, K00–K22, K29–K31, K50–K66, K71–K72, K75–K76, K83–M99, N13.0–N13.5, N13.7–N13.9, N14, N15.0, N15.8–N15.9, N20–N23, N28–N39, N41–N64, N80–N98	18,946	1.00 (0.98–1.03)	608	1.03 (0.86–1.23)
114	Motor vehicle accidents	V02–V04, V09.0, V09.2, V12–V14, V19.0–V19.2, V19.4–V19.6, V20–V79, V80.3–V80.5, V81.0–V81.1, V82.0–V82.1, V83–V86, V87.0–V87.8, V88.0–V88.8, V89.0, V89.2	658	0.91 (0.81–1.03)	31	1.08 (0.45–2.61)
118	Falls	W00–W19	2739	0.97 (0.91–1.04)	62	1.17 (0.67–2.06)
122	Accidental poisoning and exposure to noxious substances	X40–X49	224	0.92 (0.74–1.13)	15	3.49 (0351–23.97)
123	Other and unspecified non-transport accidents	W20–W31, W35–W64, W75–W99, X10–X39, X50–X59, Y86	942	1.06 (0.95–1.18)	47	1.76 (0.74–4.22)

^1^ Odds ratios reflect increase in risk associated with each 1 µg/m^3^ increase in average PM_2.5_ in the 365 days preceding cases’ death dates. Models were adjusted for age, sex, race, dual-Medicaid eligibility; zip code-level annual average NO_2_ and O_3_, SVI, metropolitan residence, percent white, black, Hispanic and Native American residents; percent of residents with less than a high school education; percent of residents 65 and older living below the poverty level; percent of owner-occupied homes, and month of case’s death. Exception: models for Native Americans did not include race.

## Data Availability

Restrictions apply to the availability of Medicare beneficiary data which was obtained by the authors from the Centers for Medicare and Medicaid Services, and subject to a Data Use Agreement.

## Supplementary Materials

The following supporting information can be downloaded at https://www.mdpi.com/article/10.3390/ijerph22091340/s1, Figure S1: Study population flow chart; Table S1: Characteristics of the study population, by decile; Table S2: Median ambient PM2.5 exposure by race; Table S3: Associations between PM2.5 and mortality, full study population and population excluding Native Americans, by decile; Table S4: Socioeconomic status indicators within each beneficiary race category, decile 1; Table S5: Comparison of zip code-specific PM2.5 concentrations modeled by SEDAC and measured by EPA, select monitors in rural Arizona; Table S6: State of residence, cases and controls in decile 1 overall and decile 1 Native Americans.

## Abbreviations

The following abbreviations are used in this manuscript:

PM_2.5_	Fine particulate matter
HR	Hazard ratio
EPA	U.S. Environmental Protection Agency
NAAQS	National Ambient Air Quality Standard
CRF	Concentration response function
CMS	Centers for Medicare and Medicaid Services
MBSF	Master Beneficiary Summary File
ICD-10	International Classification of Diseases Tenth Revision
NO_2_	Nitrogen dioxide
O_3_	Ozone
SEDAC	Socioeconomic Data and Applications Center
SVI	Social vulnerability index
CDC	U.S. Centers for Disease Control and Prevention
RUCA	Rural-Urban Commuting Area
SES	Socioeconomic status
NAN	North American Native
OR	Odds ratio
USD	US dollar
COPD	Chronic obstructive pulmonary disease
SDOH	Social determinants of health
IHS	Indian Health Service

## References

[1] Health Effects Institute (2016). New HEI Program to Examine Potential Health Effects at Low-Levels of Air Pollution.

[2] Shi L., Zanobetti A., Kloog I., Coull B.A., Koutrakis P., Melly S.J., Schwartz J.D. (2016). Low-Concentration PM2.5 and Mortality: Estimating Acute and Chronic Effects in a Population-Based Study. Environ. Health Perspect..

[3] Di Q., Wang Y., Zanobetti A., Wang Y., Koutrakis P., Choirat C., Dominici F., Schwartz J.D. (2017). Air Pollution and Mortality in the Medicare Population. N. Engl. J. Med..

[4] Wu X., Braun D., Schwartz J., Kioumourtzoglou M.A., Dominici F. (2020). Evaluating the impact of long-term exposure to fine particulate matter on mortality among the elderly. Sci. Adv..

[5] Wei Y., Yazdi M.D., Di Q., Requia W.J., Dominici F., Zanobetti A., Schwartz J. (2021). Emulating causal dose-response relations between air pollutants and mortality in the Medicare population. Environ. Health Glob. Access Sci. Source.

[6] Dominici F., Zanobetti A., Schwartz J., Braun D., Sabath B., Wu X. (2022). Assessing Adverse Health Effects of Long-Term Exposure to Low Levels of Ambient Air Pollution: Implementation of Causal Inference Methods. Res. Rep..

[7] U.S. Environmental Protection Agency (2024). Final Rule to Strengthen the National Air Quality Health Standard for Particulate Matter.. https://www.epa.gov/system/files/documents/2024-02/pm-naaqs-overview.pdf.

[8] Schwartz J., Wei Y., Yitshak-Sade M., Di Q., Dominici F., Zanobetti A. (2021). A national difference in differences analysis of the effect of PM2.5 on annual death rates. Environ. Res..

[9] Chen J., Hoek G. (2020). Long-term exposure to PM and all-cause and cause-specific mortality: A systematic review and meta-analysis. Environ. Int..

[10] World Health Organization (2019). International Classification of Diseases Tenth Revision (ICD-10).

[11] Wei Y., Xing X., Shtein A., Castro E., Hultquist C., Yazdi M.D., Li L., Schwartz J. (2022). Daily and Annual PM2.5, O3, and NO2 Concentrations at ZIP Codes for the Contiguous US, 2000-2016, v1.0 (Version 1.00) [Data Set].

[12] Di Q., Amini H., Shi L., Kloog I., Silvern R., Kelly J., Sabath M.B., Choirat C., Koutrakis P., Lyapustin A. (2019). An ensemble-based model of PM2.5 concentration across the contiguous United States with high spatiotemporal resolution. Environ. Int..

[13] Thornton M.M., Shrestha R., Wei Y., Thornton P.E., Kao S.C. (2022). Daymet: Daily Surface Weather Data on a 1-km Grid for North America, Version 4 R1 (Version 4.1).

[14] Center for International Earth Science Information Network, (CIESIN), Columbia University (2023). U.S. Social Vulnerability Index Grids, Revision 01 (Version 1.01) [Data Set].

[15] U.S. Department of Agriculture Economic Research Service (2025). Rural-Urban Commuting Area Codes—Documentation.. https://www.ers.usda.gov/data-products/rural-urban-commuting-area-codes/documentation.

[16] U.S. Census Bureau Explore Census Data.. https://data.census.gov/.

[17] Bowe B., Xie Y., Yan Y., Al-Aly Z. (2019). Burden of Cause-Specific Mortality Associated with PM2.5 Air Pollution in the United States. JAMA Netw. Open.

[18] Zhao X., Wu T., Zhou W., Han L., Neophytou A.M. (2024). Reducing air pollution does not necessarily reduce related adults’ mortality burden: Variations in 177 countries with different economic levels. Sci. Total Environ..

[19] Bunnell J.E., Garcia L.V., Furst J.M., Lerch H., Olea R.A., Suitt S.E., Kolker A. (2010). Navajo coal combustion and respiratory health near Shiprock, New Mexico. J. Environ. Public Health.

[20] Seltenrich N. (2012). Healthier tribal housing: Combining the best of old and new. Environ. Health Perspect..

[21] Hadeed S.J., O’Rourke M.K., Canales R.A., Joshweseoma L., Sehongva G., Paukgana M., Gonzalez-Figueroa E., Alshammari M., Burgess J.L., Harris R.B. (2021). Household and behavioral determinants of indoor PM2.5 in a rural solid fuel burning Native American community. Indoor Air.

[22] Office of Disease Prevention and Health Promotion, U.S. Department of Health and Human Services Social Determinants of Health. https://odphp.health.gov/healthypeople/priority-areas/social-determinants-health.

[23] Jones D.S. (2006). The persistence of American Indian health disparities. Am. J. Public Health.

[24] Arias E., Xu J., Curtin S., Bastian B., Tejada-Vera B. (2021). Mortality Profile of the Non-Hispanic American Indian or Alaska Native Population, 2019. Natl. Vital Stat. Rep..

[25] Medicaid and CHIP Payment and Access Commission (MACPAC) (2021). Issue Brief: Medicaid’s Role in Health Care for American Indians and Alaska Natives.

[26] Odani S., Armour B.S., Graffunder C.M., Garrett B.E., Agaku I.T. (2017). Prevalence and Disparities in Tobacco Product Use Among American Indians/Alaska Natives—United States, 2010–2015. MMWR. Morb. Mortal. Wkly. Rep..

[27] Arrazola R.A., Griffin T., Lunsford N.B., Kittner D., Bammeke P., Courtney-Long E.A., Armour B.S. (2023). US Cigarette Smoking Disparities by Race and Ethnicity—Keep Going and Going!. Prev. Chronic Dis..

[28] Azagba S., Shan L., Latham K., Qeadan F. (2020). Trends in cigarette smoking among American Indians and Alaska Natives in the USA: 1992–2015. Cancer Causes Control.

[29] U.S. Environmental Protection Agency (2019). Integrated Science Assessment (ISA) for Particulate Matter (Final Report, December 2019).

[30] Boccuti C., Swoope C., Artiga S. (2014). The Role of Medicare and the Indian Health Service for American Indians and Alaska Natives: Health, Access and Coverage.

[31] United States Government Accountability Office (2005). Indian Health Service: Health Care Services Are Not Always Available to Native Americans.

[32] Kaiser Family Foundation Status of State Medicaid Expansion Decisions. https://www.kff.org/status-of-state-medicaid-expansion-decisions/.

[33] Hajat A., MacLehose R.F., Rosofsky A., Walker K.D., Clougherty J.E. (2021). Confounding by Socioeconomic Status in Epidemiological Studies of Air Pollution and Health: Challenges and Opportunities. Environ. Health Perspect..

[34] Luo J., Craver A., Jin Z., Zheng L., Kim K., Polonsky T., Olopade C.O., Pinto J.M., Ahsan H., Aschebrook-Kilfoy B. (2024). Contextual Deprivation, Race and Ethnicity, and Income in Air Pollution and Cardiovascular Disease. JAMA Netw. Open.

[35] Singh G., Williams S., Lee H., Martin E., Allender M., Ramey C. (2021). Trends in Physical and Mental Health, Mortality, Life Expectancy, and Social Inequalities Among American Indians and Alaska Natives, 1990–2019. Int. J. Transl. Med. Res. Public Health.

[36] Li M., Hilpert M., Goldsmith J., Brooks J.L., Shearston J.A., Chillrud S.N., Ali T., Umans J.G., Best L.G., Yracheta J. (2022). Air Pollution in American Indian Versus Non-American Indian Communities, 2000–2018. Am. J. Public Health.

[37] Li M., Do V., Brooks J.L., Hilpert M., Goldsmith J., Chillrud S.N., Ali T., Best L.G., Yracheta J., Umans J.G. (2023). Fine particulate matter composition in American Indian vs. Non-American Indian communities. Environ. Res..

[38] Nunez Y., Benavides J., Shearston J.A., Krieger E.M., Daouda M., Henneman L.R.F., McDuffie E.E., Goldsmith J., Casey J.A., Kioumourtzoglou M.-A. (2024). An environmental justice analysis of air pollution emissions in the United States from 1970 to 2010. Nat. Commun..

[39] Henneman L., Choirat C., Dedoussi I., Dominici F., Roberts J., Zigler C. (2023). Mortality risk from United States coal electricity generation. Science.

[40] Henneman L.R.F., Rasel M.M., Choirat C., Anenberg S.C., Zigler C. (2023). Inequitable Exposures to U.S. Coal Power Plant-Related PM2.5: 22 Years and Counting. Environ. Health Perspect..

[41] Swanson A., Holden Z.A., Graham J., Warren D.A., Noonan C., Landguth E. (2022). Daily 1 km terrain resolving maps of surface fine particulate matter for the western United States 2003–2021. Sci. Data.

[42] U.S. Environmental Protection Agency Air Data: Air Quality Data Collected at Outdoor Monitors Across the U.S.. https://www.epa.gov/outdoor-air-quality-data.

[43] Jbaily A., Zhou X., Liu J., Lee T.-H., Kamareddine L., Verguet S., Dominici F. (2022). Air pollution exposure disparities across US population and income groups. Nature.

[44] Josey K.P., Delaney S.W., Wu X., Nethery R.C., DeSouza P., Braun D., Dominici F. (2023). Air Pollution and Mortality at the Intersection of Race and Social Class. N. Engl. J. Med..

[45] Loccoh E., Joynt Maddox K.E., Xu J., Shen C., Figueroa J.F., Kazi D.S., Yeh R.W., Wadhera R.K. (2021). Rural-Urban Disparities In All-Cause Mortality Among Low-Income Medicare Beneficiaries, 2004–2017. Health Aff..

[46] Danesh Yazdi M., Amini H., Wei Y., Castro E., Shi L., Schwartz J.D. (2024). Long-term exposure to PM2.5 species and all-cause mortality among Medicare patients using mixtures analyses. Environ. Res..

[47] Eberly L.A., Shultz K., Merino M., Brueckner M.Y., Benally E., Tennison A., Biggs S., Hardie L., Tian Y., Nathan A.S. (2023). Cardiovascular Disease Burden and Outcomes Among American Indian and Alaska Native Medicare Beneficiaries. JAMA Netw. Open.

[48] Zhao G., Hsia J., Vigo-Valentín A., Garvin W.S., Town M. (2022). Health-Related Behavioral Risk Factors and Obesity Among American Indians and Alaska Natives of the United States: Assessing Variations by Indian Health Service Region. Prev. Chronic Dis..

[49] Jacobs-Wingo J.L., Espey D.K., Groom A.V., Phillips L.E., Haverkamp D.S., Stanley S.L. (2016). Causes and Disparities in Death Rates Among Urban American Indian and Alaska Native Populations, 1999–2009. Am. J. Public Health.

[50] Melkonian S.C., Weir H.K., Jim M.A., Preikschat B., Haverkamp D., White M.C. (2021). Incidence of and Trends in the Leading Cancers With Elevated Incidence Among American Indian and Alaska Native Populations, 2012–2016. Am. J. Epidemiol..

[51] Villarroel M.A., Clarke T.C., Norris T. (2020). Health of American Indian and Alaska Native Adults, by Urbanization Level: United States, 2014–2018. NCHS Data Brief.

[52] Park-Lee E., Lipari R.N., Bose J., Hughes A., Greenway K., Glasheen C., Herman-Stahl M., Penne M., Pemberton M., Cajka J. (2018). Substance Use and Mental Health Issues Among U.S. Born American Indians or Alaska Natives Residing on and off Tribal Lands. https://www.samhsa.gov/data/sites/default/files/cbhsq-reports/DRAIANTribalAreas2018/DRAIANTribalAreas2018.pdf.

[53] Office of Inspector General (2022). Data Brief: Inaccuracies in Medicare’s Race and Ethnicity Data Hinder the Ability to Assess Health Disparities.

[54] Jarrín O.F., Nyandege A.N., Grafova I.B., Dong X., Lin H. (2020). Validity of Race and Ethnicity Codes in Medicare Administrative Data Compared with Gold-Standard Self-Reported Race Collected During Routine Home Health Care Visits. Med. Care.

[55] Centers for Medicare and Medicaid Services (2017). Report to Congress: Improving Medicare Post-Acute Care Transformation (IMPACT) Act of 2014 Strategic Plan for Accessing Race and Ethnicity Data.

[56] Mork D., Delaney S., Dominici F. (2024). Policy-induced air pollution health disparities: Statistical and data science considerations. Science.

