# Supplementary material for: Low-Level PM2.5 Exposure and Mortality in the Medicare Cohort: The Role of Native American Beneficiaries
Source: Int J Environ Res Public Health. 2025 Aug 27;22(9):1340. doi: 10.3390/ijerph22091340 (PMC12469847; doi:10.3390/ijerph22091340)

Supplemental Material

Table S1. Characteristics of the study population, by decile

Table S2. Median ambient $PM_{2.5}$ exposure by race

Table S3. Associations between $PM_{2.5}$ and mortality, full study population and population excluding Native Americans, by decile

Table S4. Socioeconomic status indicators within each beneficiary race category, decile 1

Table S5. Comparison of zip code-specific $PM_{2.5}$ concentrations modeled by SEDAC and measured by EPA, select monitors in rural Arizona

Table S6. State of residence, cases and controls in decile 1 overall and decile 1 Native Americans

Figure S1. Study population flow chart

Table S1. Characteristics of the study population, by decile

Characteristics	Decedent Cases by Decile, No., (%)										All Decedent Cases, No. (%) (n=2,773,647)	All Non-decedent Controls, No. (%) (n=27,736,470)
	1 (n=153,781)	2 (n=220,953)	3 (n=253,816)	4 (n=283,468)	5 (n=290,231)	6 (n=299,886)	7 (n=305,616)	8 (n=311,828)	9 (n=325,526)	10 (n=328,542)		
**Beneficiary Level Covariates**												
Age, mean (SD), y	81.8 (9.3)	82.5 (9.2)	82.5 (9.24)	82.3 (9.25)	82.1 (9.26)	82.0 (9.26)	81.9 (9.26)	81.9 (9.25)	82.0 (9.25)	82.1 (9.32)	82.1 (9.3)	74.9 (8.1)
Sex												
Male	76,972 (50.1)	106,117 (48.0)	121,072 (47.7)	133,175 (47.0)	134,737 (46.4)	138,792 (46.3)	140,722 (46.1)	142,784 (45.8)	148,789 (45.7)	150,965 (46.0)	1,294,125 (46.7)	12,577,617 (45.4)
Female	76,809 (50.0)	114,836 (52.0)	132,744 (52.3)	150,293 (53.0)	155,494 (53.6)	161,094 (53.7)	164,894 (54.0)	169,044 (54.2)	176,737 (54.3)	177,577 (54.1)	1,479,522 (53.3)	15,158,850 (54.7)
Race												
White	141,834 (92.2)	206,787 (93.6)	231,805 (91.3)	252,656 (89.1)	253,981 (87.5)	256,338 (85.5)	256,019 (83.8)	258,685 (83.0)	270,208 (83.0)	262,936 (80.0)	2,391,249 (86.2)	23,222,339 (83.7)
Black	1,589 (1.0)	5,020 (2.3)	10,620 (4.2)	16,902 (6.0)	22,891 (7.9)	29,775 (9.9)	35,371 (11.6)	38,326 (12.3)	40,270 (12.4)	40,081 (12.2)	240,845 (8.7)	2,293,857 (8.3)
Other	1,269 (0.8)	1,915 (0.9)	2,341 (0.9)	2,685 (1.0)	2,818 (1.0)	3,017 (1.0)	2,998 (1.0)	3,044 (1.0)	3,162 (1.0)	4,897 (1.5)	28,146 (1.0)	480,618 (1.7)
Asian	926 (0.6)	1,708 (0.8)	2,743 (1.1)	3,384 (1.2)	3,794 (1.3)	4,113 (1.4)	4,328 (1.4)	4,921 (1.6)	5,106 (1.6)	9,261 (2.8)	40,284 (1.5)	604,350 (2.2)
Hispanic	2,732 (1.8)	2,868 (1.3)	3,936 (1.6)	4,943 (1.7)	3,953 (1.4)	3,995 (1.3)	4,249 (1.4)	4,596 (1.5)	4,952 (1.5)	9,504 (2.9)	45,728 (1.7)	515,222 (1.9)
Native American	4,709 (3.1)	1,553 (0.7)	1,079 (0.4)	1,472 (0.5)	1,371 (0.5)	1,258 (0.4)	1,189 (0.4)	861 (0.3)	369 (0.1)	356 (0.1)	14,217 (0.5)	122,238 (0.4)
Unknown	722 (0.5)	1,102 (0.5)	1,292 (0.5)	1,426 (0.5)	1,423 (0.5)	1,390 (0.5)	1,462 (0.5)	1,395 (0.5)	1,459 (0.5)	1,507 (0.5)	13,178 (0.5)	497,846 (1.8)
Dual Medicaid eligibility	37,013 (24.1)	58,710 (26.6)	68,437 (27.0)	78,822 (27.8)	80,563 (27.8)	84,255 (28.1)	87,880 (28.8)	89,287 (28.6)	90,103 (27.7)	98,445 (30.0)	773,515 (27.9)	3,669,176 (13.2)
**Zip Code-Level Covariates**												
Urbanicity												
Metropolitan	72,010 (46.8)	151,384 (68.5)	193,126 (76.1)	215,107 (75.9)	219,386 (75.6)	228,059 (76.1)	231,814 (75.9)	243,957 (78.2)	269,029 (82.7)	286,082 (87.1)	2,109,954 (76.1)	21,704,297 (78.3)
Micropolitan	36,708 (23.9)	32,274 (14.6)	27,358 (10.8)	33,658 (11.9)	36,399 (12.5)	37,217 (12.4)	38,562 (12.6)	38,367 (12.3)	34,631 (10.6)	27,439 (8.4)	342,613 (12.4)	3,113,969 (11.2)
Small Town	22,790 (14.8)	16,148 (7.3)	16,280 (6.4)	19,838 (7.0)	21,616 (7.5)	22,846 (7.6)	23,969 (7.8)	21,025 (6.7)	15,557 (4.8)	11,084 (3.4)	191,153 (6.9)	1,704,315 (6.1)
Rural	22,247 (14.5)	21,140 (9.6)	17,045 (6.7)	14,856 (5.2)	12,819 (4.4)	11,746 (3.9)	11,254 (3.7)	8,453 (2.7)	6,286 (1.9)	3,926 (1.2)	129,772 (4.7)	1,212,823 (4.4)
Social Vulnerability Index, mean (SD)	0.45 (0.26)	0.40 (0.25)	0.43 (0.25)	0.45 (0.26)	0.45 (0.27)	0.46 (0.27)	0.47 (0.27)	0.47 (0.28)	0.45 (0.28)	0.47 (0.28)	0.45 (0.27)	0.43 (0.27)
Percent White	86.4	86.7	83.7	81.3	78.6	75.9	74.1	73.4	73.8	71.9	77.7	77.6
Percent Black	1.7	4.2	6.5	8.6	10.7	13.2	15.2	16.0	15.9	14.4	11.5	11.0
Percent Hispanic	14.9	11.6	12.5	12.4	11.4	11.5	11.6	12.0	11.8	17.8	12.7	13.0
Percent Native American	3.8	1.1	0.7	0.8	0.8	0.7	0.7	0.5	0.3	0.3	0.8	0.8
Median household income, $	54,774	59,754	60,188	60,059	59,603	58,410	57,091	57,182	58,454	56,990	58 327	61 047
Median value owner-occupied housing, $	241,175	247,127	244,901	243,126	234,689	224,461	218,089	217,990	213,999	219,329	228 770	246 680
Percent of elderly below poverty level	8.6	8.2	8.6	9.0	9.2	9.6	9.9	10.0	9.6	10.2	9.4	9.0
Percent w/ less than high school education	11.1	10.8	11.3	12.1	12.4	12.9	13.3	13.	12.8	14.3	12.6	12.1
Percent of owner-occupied housing	70.4	70.4	69.1	68.5	67.5	66.7	65.6	65.0	65.2	62.6	66.7	67.2
Population density, No.	433	1,218	1,798	2,383	2,666	3,098	3,317	3,408	3 224	4 091	2 756	2 966
Annual $PM_{2.5}$, mean (SD)	3.70 (0.74)	5.29 (0.31)	6.19 (0.22)	6.86 (0.18)	7.41 (0.15)	7.88 (0.13)	8.31 (0.13)	8.81 (0.16)	9.45 (0.22)	10.79 (1.04)	7.81 (1.87)	7.81 (1.88)
Annual NO_2, mean (SD)	11.00 (5.09)	12.62 (5.93)	13.84 (6.96)	15.34 (7.95)	15.56 (8.04)	15.63 (7.92)	15.48 (7.70)	15.85 (7.37)	17.05 (7.09)	20.07 (7.07)	15.63 (7.61)	15.93 (7.72)
Annual O_3, mean (SD)	41.98 (5.46)	38.93 (4.21)	38.43 (3.70)	38.57 (3.37)	38.60 (2.90)	38.67 (2.76)	38.59 (2.68)	38.48 (2.78)	38.35 (2.65)	38.52 (3.88)	38.75 (3.48)	38.80 (3.59)

Table S2. Median[a] ambient $PM_{2.5}$ exposure by race

Characteristic	Decile 1, μg/m^3, Median	All deciles, μg/m^3, Median
All	3.81	7.93
White	3.82	7.84
Black	4.01	8.51
Hispanic	3.88	8.24
Native American	3.26	6.42

[a] Calculated as the median of daily average $PM_{2.5}$ concentrations for the 365 days prior to cases' date of death

Table S3. Associations between $PM_{2.5}$ and mortality, full study population and population excluding Native Americans, by decile

Decile	Odds ratio (95% CI)[a]			
	All subjects		Native American subjects excluded	
	Unadjusted	Adjusted	Unadjusted	Adjusted
1	1.060 (1.052-1.067)	1.013 (1.005-1.022)	1.061 (1.054-1.069)	1.009 (1.000-1.018)
2	1.025 (1.011-1.039)	1.016 (1.001-1.032)	1.025 (1.011-1.039)	1.015 (1.000-1.031)
3	1.016 (0.997-1.035)	1.008 (0.988-1.029)	1.015 (0.996-1.034)	1.008 (0.988-1.029)
4	1.045 (1.023-1.069)	1.044 (0.992-1.049)	1.046 (1.023-1.069)	1.044 (1.020-1.069)
5	1.021 (0.995-1.048)	1.020 (0.995-1.052)	1.022 (0.996-1.049)	1.019 (0.991-1.048)
6	1.027 (0.997-1.058)	1.041 (1.009-1.075)	1.028 (0.997-1.059)	1.042 (1.010-1.075)
7	1.016 (0.987-1.046)	1.014 (0.984-1.046)	1.016 (0.987-1.046)	1.014 (0.983-1.045)
8	0.986 (0.964-1.009)	1.004 (0.980-1.029)	0.985 (0.963-1.008)	1.002 (0.978-1.027)
9	0.992 (0.976-1.009)	0.997 (0.979-1.015)	0.992 (0.976-1.009)	0.997 (0.980-1.015)
10	0.987 (0.984-0.991)	0.996 (0.992-1.000)	0.987 (0.983-0.990)	0.996 (0.991-1.000)

[a] Odds ratios reflect increase in risk associated with each 1 $\mu g/m^3$ increase in average $PM_{2.5}$ in the 365 days preceding cases' death dates. Models adjusted for age, sex, race, dual-Medicaid eligibility; zip code-level annual average NO_2 and O_3, SVI, metropolitan residence, percent white, black, Hispanic and Native American residents; percent of residents with less than a high school education; percent of residents 65 and older living below the poverty level; percent of owner-occupied homes, and month of case's death.

Table S4. Socioeconomic status indicators within each beneficiary race category, decile 1

Indicator	White	Black	Hispanic	Native American
Dual Medicaid eligibility, No. (%)	156,717 (10.2)	4,005 (23.5)	14,140 (52.3)	18,320 (41.5)
Median household income, $	52,193	53,236	47,733	34,352
Median value owner-occupied housing	218,600	193,500	174,600	105,400
Percent of elderly below poverty level	7.8	9.8	11.7	22.8
Percent w/ less than high school education	10.1	13.5	20.2	20.2
Percent of owner-occupied housing	71.4	64.8	64.4	70.5

Table S5. State of residence, cases and controls in decile 1 overall and decile 1 restricted to Native American beneficiaries

State of Residence	Decile 1 – All, No., (%)		Decile 1 - Native Americans, No., (%)	
	Cases	Controls	Cases	Controls
AZ	20,989 (13.7)	217,965 (14.2)	1,478 (31.4)	13,759 (34.9)
CA	21,779 (14.2)	220,278 (14.3)	358 (7.6)	2,476 (6.3)
CO	9,854 (6.4)	116,662 (7.6)	60 (1.3)	443 (1.1)
CT	145 (0.1)	1,373 (0.1)		
ID	4,390 (2.9)	43,433 (2.8)	29 (0.6)	254 (0.6)
MD	54 (0.0)	509 (0.0)		
ME	5,256 (3.4)	43,271 (2.8)	31 (0.7)	224 (0.6)
MI	1,414 (0.9)	12,435 (0.8)	27 (0.6)	307 (0.8)
MN	1,865 (1.2)	16,633 (1.1)	114 (2.4)	793 (2.0)
MO	16 (0.0)	162 (0.0)		
MT	4,668 (3.0)	4,3261 (2.8)	179 (3.8)	1,365 (4.5)
NC	1,166 (0.8)	10,259 (0.7)	76 (1.6)	457 (1.2)
ND	1,210 (0.8)	10,658 (0.7)	90 (1.9)	653 (1.7)
NE	1,359 (0.9)	10,943 (0.7)	12 (0.3)	84 (0.2)
NM	11,645 (7.6)	116,654 (7.6)	1,222 (26.0)	10,824 (27.4)
NV	7,453 (4.9)	75,929 (4.9)	85 (1.8)	900 (2.3)
NY	3,244 (2.1)	28,594 (1.9)	31 (0.7)	269 (0.7)
OR	6,446 (4.2)	65,505 (4.3)	97 (2.1)	789 (2.0)
SD	2,709 (1.8)	23,997 (1.6)	332 (7.1)	2,206 (5.6)
TN	26 (0.0)	168 (0.0)		
UT	7,175 (4.7)	69,960 (4.6)	104 (2.2)	715 (1.8)
WA	15,394 (10.0)	159,247 (10.4)	240 (5.1)	1,889 (4.8)
WI	817 (0.5)	7,695 (0.5)	41 (0.9)	249 (0.6)
WY	6,147 (4.0)	60,750 (4.0)	83 (1.8)	660 (1.7)

Table S6. Comparison of zip code-specific $PM_{2.5}$ concentrations modeled by SEDAC and measured by EPA[a], select monitors in rural Arizona

		**2015**					**2016**				
		**Annual Average[b], μg/m³**		**Annual 24-hr Maximum, μg/m³**			**Annual Average[b], μg/m³**		**Annual 24-hr Maximum, μg/m³**		
**EPA Monitor**	**County (zip code)**	**Measured - EPA (No.[c])**	**Modeled - SEDAC**	**Date - EPA**	**Measured - EPA**	**Modeled - SEDAC**	**Measured - EPA (No.[c])**	**Modeled - SEDAC**	**Date(s) - EPA**	**Measured - EPA**	**Modeled - SEDAC**
04-001-1235-1[d]	Apache (86503)	2.7 (48)	2.9	6/23/2015	6.5	7.1	2..4 (45)	3.0	6/5/2016	8.7	5.2
04-001-1235-2[d]	Apache (86503)	2.8 (48)	2.9	6/23/2015	6.4	7.1	2..4 (45)	3.0	6/5/2016	8.7	5.2
04-013-7020-1[e]	Maricopa (85256)	4.7 (47)	6.5	11/26/2015	8.6	12.1	8.5 (60)	6.5	1/1/2016	28.6	68.5
04-013-7020-2[e]	Maricopa (85256)	4.8 (57)	6.5	11/26/2015	8.5	12.1	7.6 (51)	6.5	1/1/2016	28.1	68.5
04-015-1000-1[f]	Mohave (86434)	4.6 (43)	2.6	4/6/2015	11.1	4.4	4.9 (31)	2.8	12/2/2016	21.0	1.7
04-021-0001-1	Pinal (85122)	6.7 (105)	6.6	12/29/2015	21.3	8.5	8.6 (119)	6.6	7/29/2016	33.7	6.7
04-021-0001-3	Pinal (85122)	7.7 (337)	6.6	12/5/2015	21.0	10.0	8.9 (345)	6.6	3/7/2016, 7/22/2016	20.6	10.4/9.0
04-021-3002-1	Pinal (85119)	4.8 (116)	4.7	11/26/2015	11.3	7.5	5.2 (117)	4.3	3/7/2016	14.6	8.9
04-021-3013-1	Pinal (85138)	10.2 (117)	8.5	12/2/2015	27.1	12.7	N/A	--	N/A	N/A	--
04-021-3013-2	Pinal (85138)	9.7 (58)	8.5	12/2/2015	27.5	12.7	N/A	--	N/A	N/A	--
04-021-3015-1	Pinal (85138)	N/A	--	N/A	N/A	--	14.0 (112)	7.2	7/29/2016	64.5	10.0
04-021-3015-2	Pinal (85138)	N/A	--	N/A	N/A	--	13.6 58)	7.2	7/29/2016	62.5	10.0
04-021-3015-3	Pinal (85138)	N/A	--	N/A	N/A	--	11.3 (333)	7.2	6/23/2016	46.2	16.9
04-023-0004-1	Santa Cruz (85621)	7.7 (54)	3.7	1/6/2015	22.6	2.1	9.9 (58)	4.7	1/1/2016	105.0	104.5
04-023-0004-2	Santa Cruz (85621)	7.6 (25)	3.7	1/6/2015	22.1	2.1	N/A	--	N/A	N/A	--
04-023-0004-3	Santa Cruz (85621)	9.0 (355)	3.7	12/31/2015	49.4	10.9	9.8 (347)	4.7	N/A	133.1	104.5
04-027-8011-3	Yuma (85365)	5.6 (306)	6.3	12/26/2015	30.3	8.6	8.3 (350)	6.2	7/30/2016	44.7	9.8

[a] Source: EPA Air Data pre-generated data files, "Table of Annual Summary Data" for annual average at each monitor, and "Table of Daily and Daily Summary Data" for annual 24-hr maximum at each monitor
[b] Average of 24-hr measurements collected at the monitoring site
[c] Number of days during the calendar year that measurements were collected at the monitoring site
[d] Tribal monitoring site: Navajo Nation, AZ, NM, UT
[e] Tribal monitoring site: Salt River Pima-Maricopa Indian Community of Salt River Reservation, AZ
[f] Tribal monitoring site: Hua lapai Indian Tribe of Hualapai Indian Reservation, AZ

Figure S1. Study population flow chart

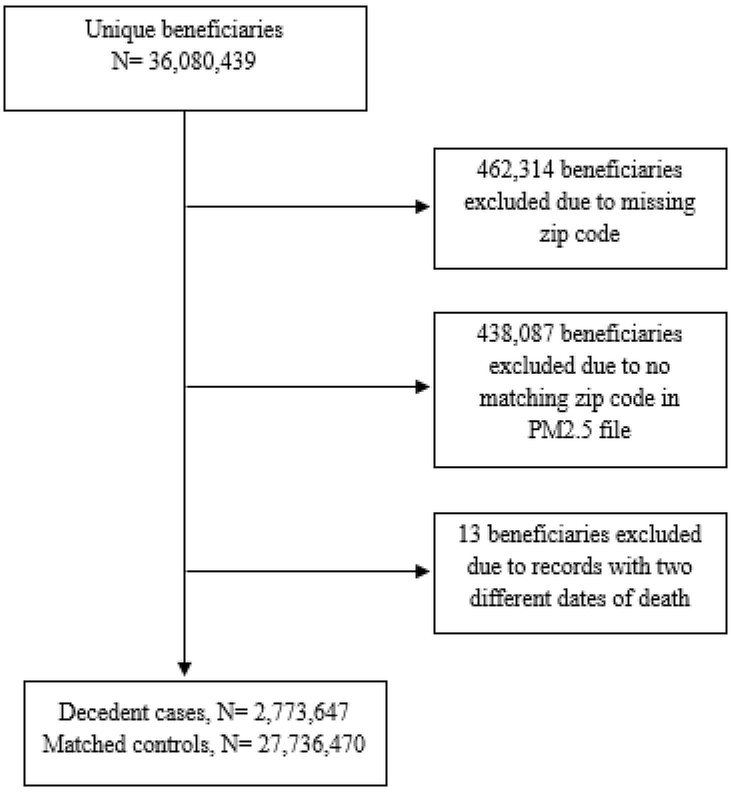

Supplement: Supplementary file 1 [file ijerph-22-01340-s001.zip › ijerph-3721423-supplementary.pdf]
